# Burnout and Intention to Change Profession among Romanian Dentists during COVID-19: A Cross Sectional Study Using the Maslach Burnout Inventory

**DOI:** 10.3390/healthcare11192667

**Published:** 2023-10-01

**Authors:** Ioana Silistraru, Anamaria Ciubară, Oana Olariu, Ioan-Adrian Ciureanu, Laura-Elisabeta Checheriță, Daniela Drugus, Radu Dănilă, Ștefan Roșca

**Affiliations:** 1 Faculty of Social Sciences and Humanities, Lucian Blaga University of Sibiu, 550025 Sibiu, Romania; ioana.silistraru@ulbsibiu.ro; 2 Clinical Medical Department, School of Medicine and Pharmacy, Dunărea de Jos University of Galați, 800008 Galati, Romania; 3 Grigore T. Popa University of Medicine and Pharmacy, 700115 Iasi, Romania; adrian.ciureanu@umfiasi.ro (I.-A.C.); laura.checherita@umfiasi.ro (L.-E.C.); daniela.drugus@umfiasi.ro (D.D.);

**Keywords:** burnout, dentistry, COVID-19, mental health, healthcare

## Abstract

This study aims to examine the relationship between burnout and dentists’ intentions to change careers during the COVID-19 pandemic. The MBI-Human Services Survey for Medical Personnel—MBI-HSS (MP) was used to measure burnout levels and investigate how they relate to dentists’ intentions to change their profession. The sample included 69 Romanian dentists, 56 of whom were women and 13 of whom were men. Self-reported questionnaires provided to the participants were used to collect the data. Female participants reported higher levels of emotional Exhaustion and Depersonalisation than males. However, there were no substantial differences in Personal Accomplishment levels between genders. As for the intentions to change careers, 41 expressed a clear intention to change their profession, 15 were still determining if they would choose the same speciality, and 28 indicated they would not choose the medical field. The study’s findings provide insight into how dentists’ thoughts about perceived burnout during the COVID-19 pandemic significantly influenced their attitudes regarding their career paths. The results suggest concerns regarding burnout in the dental field and emphasise the necessity for burnout interventions and support services, particularly during healthcare crises. Further research and interventions to mitigate burnout and promote well-being among dentists are needed to ensure the sustainability and quality of dental healthcare services in Romania.

## 1. Introduction

The well-being and mental health of dental practitioners have been significantly impacted by the COVID-19 epidemic, along with other aspects of healthcare [1,2,3,4]. Burnout among dental professionals is rising globally because of demanding work conditions, increasing workload, and elevated risk of virus infection during the COVID-19 pandemic. Burnout, a multidimensional phenomenon [5,6,7,8] marked by emotional Exhaustion (EE), Depersonalisation (D), and diminished Personal Accomplishment (PA), can have a negative impact on dental professionals’ physical and mental health, as well as on their ability to do work effectively and provide high-quality care to their patients [9].

This scientific study aims to explore and assess burnout levels using the MBI-Human Services Survey for Medical Personnel—MBI-HSS (MP) and examine the link between burnout and various factors, such as workload, perceived stress, and coping mechanisms, to gain a deeper understanding of the burnout concerns among dental professionals during the COVID-19 pandemic. The MBI-HSS (MP) is a widely used and validated instrument for assessing burnout in healthcare professionals, including dentistry practitioners [10,11,12].

Even before the pandemic, prior research had shown that burnout was frequently encountered among dental practitioners, with significant levels of emotional Exhaustion and Depersonalisation among dentists [10,13]. However, the COVID-19 pandemic has increased stress levels and challenges for dentists to never-encountered levels, so it is essential to investigate its impact on burnout specifically at this time.

Insights into the specific challenges dental practitioners face have been highlighted by several studies examining the psychological well-being of healthcare workers during the pandemic [1,14,15,16]. More focused research on the COVID-19 pandemic’s impact on dental professionals’ burnout is nevertheless needed, as well as on the specific interventions directed at addressing the prevalence of burnout in healthcare services.

This study intends to add to the expanding body of knowledge on burnout among dental practitioners during the COVID-19 pandemic by using the MBI-HSS(MP) and incorporating findings from additional relevant research. The findings will offer insightful information about burnout levels, risk factors, and relevant interventions that can be implemented to help dental professionals manage and prevent burnout.

## 2. Materials and Methods

### 2.1. Study Design

In this cross-sectional study, we evaluated the prevalence of burnout among Romanian dental healthcare workers using an MBI-HSS(MP) customised online questionnaire, assessing three burnout dimensions: emotional Exhaustion, Depersonalisation, and the perception of Personal Accomplishment. As the survey was voluntary and anonymous, 69 Romanian dental medical professionals were provided with the questionnaire. The questionnaire was applied based on the authorisation letter obtained by Mrs Oana Olariu from Mind Garden on 26 September 2022.

We used the Mind Garden platform to conduct the licensed survey in the original English language because we concluded that our sample group did not have any difficulties or sociocultural barriers with the usage of English. To avoid the possibility of replies being provided multiple times, we gathered the data via a licensed, one-time-only accessible questionnaire link. The sample group clicked on the link to the survey, which ensured complete anonymity and voluntary participation while obtaining informed consent and explaining the objective of data collection to respondents.

The questionnaire included demographic variables such as age and gender, as well as questions regarding the intention to change their specialty or profession based on their professional experience during the COVID-19 pandemic. The customised questionnaire items were placed after the items in the original instrument to preserve the psychometric qualities of the subscales.

The MBI-HSS(MP) instrument was used to examine how frequently the sample group expressed emotional Exhaustion, Depersonalisation, and their evaluation of their level of Personal Accomplishment when working in a pandemic scenario. While burnout is a serious issue impacting healthcare professionals worldwide, its prevalence and extent must be assessed, particularly in the demanding setting of dentistry practice. Burnout is defined in our sample group by the subscales of emotional Exhaustion, in which respondents feel overwhelmed and stressed (example scale item, “I feel emotionally drained from my work.”); Depersonalisation, in which respondents lose enthusiasm and passion for their work and patients (example scale item, “I do not really care what happens to some patients.”); and Personal Accomplishment, in which respondents perceive a reasonable level of competence and achievement in their work (example scale item, “I have accomplished many worthwhile things in this job.”). The frequency average scores of the groups were determined using a Likert scale, with responses categorised as follows: 0—never; 1—once or twice a year or less; 2—once a month or less; 3—a few times a month; 4—once a week; 5—a few times a week; and 6—every day regarding the emotional Exhaustion, Depersonalisation, and Personal Accomplishment subscales.

The descriptive statistics and bivariate correlations were obtained using IBM SPSS Statistics v.26. Chi-squared test was conducted using a significance level (α) of 0.05. Also, we conducted a linear regression analysis where the dependent variable was the intention to change profession, and the independent variables were the burnout dimensions of emotional Exhaustion (EE), Depersonalisation (D), and Personal Accomplishment (PA). The significance level was set at conventional level of *p* < 0.05.

### 2.2. Participants and Settings

Our sample group consisted of 69 dental healthcare professionals, 56 female (81.15%) and 13 male (18.84%), and N mean age = 38.79, where the mean age for female participants was 38.78 and the mean age for male participants was 38.84 (Table 1).

### 2.3. Data Collection

We collected data using the MBI-HSS(MP) customised questionnaire through the Mind Garden platform in November 2021 to measure how often respondents self-reported emotional Exhaustion, Depersonalisation, and low levels of Personal Accomplishment during their activity in the pandemic work setting. As burnout has been suggested to be prevalent in healthcare students [17,18,19,20,21] and personnel [8,9,22] during the COVID-19 pandemic, we looked into data specifically for Romanian dental healthcare professionals.

### 2.4. Ethical Considerations

All participants were informed of the purpose of the study and offered consent by clicking on the licensed questionnaire, as they were free to withdraw from the data collection at any time during the online process.

## 3. Results

The findings relate to 69 active Romanian practising dentists during the COVID-19 pandemic. The responses of the participants were examined regarding their intentions to change professions and other burnout-related variables. The Maslach Burnout Inventory-Human Services Survey (MBI-HSS) and the Medical Personnel (MP) scale assessed the burnout dimensions. Personal Accomplishment (PA), Depersonalisation (D), and emotional Exhaustion (EE) were the subscales that were analysed. The burnout experiences of the participants showed significant gender differences. In total, 71.42% of the female participants and 46.15% of the male participants indicated high degrees of emotional Exhaustion. Like Depersonalisation, women (21.42%) reported it more than men (15.38%). However, there were no obvious gender differences in the Personal Accomplishment subscale.

Also, the chi-squared test results indicate that there was a statistically significant association between gender and high emotional Exhaustion (EE), but no statistically significant associations between gender and burnout levels in the “Depersonalization (D)” and “Personal Accomplishment (PA)” subscales in our dataset (Table 2).

### 3.1. Emotional Exhaustion (EE)

Among female participants, 71.42% (40 individuals) reported high levels of emotional Exhaustion, 19.64% (11 individuals) reported moderate levels, and 8.92% (5 individuals) reported low levels.

Among male participants, 46.15% (six individuals) reported high levels of emotional Exhaustion, 15.38% (two individuals) reported moderate levels, and 38.46% (five individuals) reported low levels.

Overall, 66.66% of the participants reported high levels of emotional Exhaustion, 18.84% reported moderate levels, and 14.49% reported low levels.

The chi-squared test for the “Emotional Exhaustion” subscale suggests that there was a statistically significant association between gender and burnout levels for the “High” category (*p* = 0.024). For the “Moderate” and “Low” levels of burnout, the test did not find any statistically significant association with EE.

### 3.2. Depersonalisation (D)

Depersonalisation (D) is overall described by the presence of cynicism and disconnection from patients. According to the results, more female participants (21.42%) than male participants (15.38%) reported experiencing significant levels of Depersonalisation.

Among female participants, 21.42% (12 respondents) reported high levels of Depersonalisation, 26.78% (15 respondents) reported moderate levels, and 51.78% (29 respondents) reported low levels of Depersonalisation. Among male participants, 15.38% (two respondents) reported high levels of Depersonalisation, 23.07% (three respondents) reported moderate levels, and 61.53% (eight respondents) reported low levels of Depersonalisation.

Overall, 20.28% of the participants reported high levels of Depersonalisation, 26.08% reported moderate levels, and 53.62% reported low levels of Depersonalisation regarding their work and patients.

The chi-squared test value was 0.805, suggesting there was no statistically significant association between gender and Depersonalisation in our sample.

### 3.3. Personal Accomplishment (PA)

The Personal Accomplishment (PA) dimension refers to respondents’ perception of their level of competence and ability to perform at work successfully. According to the results, a similar percentage of male and female participants reported feeling accomplished personally. However, compared with 4 male respondents (46.15%), a slightly higher percentage of female respondents (26, 46.42%) reported low levels of Personal Accomplishment.

Overall, 28.98% of the total participants reported high levels of Personal Accomplishment, 24.63% reported moderate levels, and 46.37% reported low levels.

The chi-squared test value was 0.983, suggesting there is no statistically significant association between gender and Personal Accomplishment in our dataset.

The chi-squared test results for all burnout dimensions indicate that there was a statistically significant association between gender and high emotional Exhaustion (EE), but no statistically significant association between gender and burnout levels in the Depersonalisation (D) and Personal Accomplishment (PA) subscales in our study sample.

### 3.4. Manifesting Profession Change Intentions Regarding Burnout Dimensions

In our study on burnout among Romanian dental healthcare professionals using MBI-HSS(MP), we also explored the intention to change professions in our sample group. The choice to change professions is an essential aspect to consider, as it reflects the potential impact of burnout on career decisions within the healthcare workforce in general and dentistry. The respondents in our sample group were asked to provide an answer to the question of whether they would choose or not the same medical speciality if given a chance again or whether they were not sure about a decision at the time of questionnaire completion.

In our sample, 41 participants (59.42% of the total respondents) answered “yes”, indicating that they would choose the same speciality if they had to make the decision again. Among these 41 participants, 35 were female and 6 were male. The mean age of all participants who responded “yes” was 38.75 ± 8.12 years, with females having a slightly lower mean age of 38.65 ± 8.04 years and males having a mean age of 39.33 ± 9.35 years. Fifteen participants (21.74% of the total respondents) were uncertain and answered that they were not sure if they would choose the same speciality again. Among these 15 participants, 13 were female and 2 were male. The mean age of all participants who were uncertain was 37.06 ± 8.18 years, with females having a mean age of 36.84 ± 7.19 years and males having a mean age of 38.50 ± 17.67 years. Twenty-eight participants (18.84% of the total respondents) answered “no”, indicating that they would not choose the same medical speciality if given a choice again. Among these 28 participants, 24 were female and 4 were male. The mean age of all participants who responded “no” was 39.92 ± 8.04 years, with females having a mean age of 39.66 ± 8.12 years and males having a mean age of 41.50 ± 8.54 years.

Our data revealed that many participants intended to change their profession due to burnout. Among the participants who reported high levels of emotional Exhaustion, 50% of female participants and 33.33% of male participants expressed the intention to change their profession. Additionally, among those experiencing high levels of Depersonalisation, 41.66% of female and 50% of male participants expressed the intention to change their profession. These findings suggest that burnout may play a significant role in shaping career decisions, particularly among those experiencing higher levels of emotional Exhaustion and Depersonalisation.

The correlation matrix suggests that burnout dimensions (EE, D, PA) are related to our sample group’s intention to change from the dental profession (Table 3).

Responses in our sample group suggest that emotional Exhaustion (EE) is positively correlated to changing professions (*r* = 0.307, *p* = 0.010), indicating that higher levels of emotional Exhaustion were associated with a greater likelihood of dental practitioners considering changing their career path. Comparably, results on the Depersonalisation (D) subscale suggest a positive correlation to changing professions (*r* = 0.163, *p* = 0.181), although this correlation was not statistically significant at the conventional threshold of *p* < 0.05.

Based on the coefficients of the performed linear regression model, our data suggest that emotional Exhaustion has a moderate positive effect (Beta = 0.338) on the desire to change profession, and the p value (*p* = 0.011) indicated a statistically significant relationship. Depersonalisation provides a weak negative effect (Beta = −0.117), but the high p value (*p* = 0.489) suggests that the effect is not statistically significant. The Personal Accomplishment Beta coefficient of 0.221 indicated a moderate positive effect related to the desire to change profession; however, this is not statistically significant with a p value of 0.128 (Table 4).

## 4. Discussion

The present study aims to explore burnout dimensions, specifically focusing on dental healthcare professionals using the Maslach Burnout Inventory-Human Services Survey (MBI-HSS) with the Medical Personnel (MP) scale. Our results suggest important gender differences in the experience of burnout among dental healthcare professionals, particularly concerning the emotional Exhaustion (EE) dimension.

Emotional Exhaustion is a critical dimension within the multidimensional construct of burnout, characterised by feeling emotionally drained and overburdened facing professional duties [10,11]. The study results suggest that both male and female dental healthcare professionals experienced considerable emotional Exhaustion. However, a higher proportion of female respondents (71.42%) reported high levels of emotional Exhaustion compared with male respondents (46.15%). These results are consistent with earlier studies suggesting that factors such as workload, patient expectations, and the challenge of balancing work and family responsibilities may contribute to higher emotional Exhaustion experienced by female healthcare practitioners in general [1,8,22,23].

Depersonalisation (D) is another measured dimension of burnout, characterised by cynicism and disconnection from patients. Our findings suggest that more female participants (21.42%) reported significant levels of Depersonalisation compared with male respondents (15.38%). However, it is important to highlight that for our sample group, regardless of gender, Depersonalisation was only minimal. Our study suggests that Depersonalisation, while present to some degree, is less prominent than emotional Exhaustion, as suggested by previous studies [8,24].

Regarding the Personal Accomplishment (PA) subscale of the MBI-HSS (MP) questionnaire, which gauges a person’s perception of their competence and ability to perform satisfactorily at work, our results indicate that a similar proportion of male and female participants reported feeling very accomplished on a personal level. However, a slightly higher percentage of female respondents (46.42%) reported low levels of Personal Accomplishment compared with male respondents (46.15%). These findings raise possible concerns about challenges to the professional fulfilment of female dental healthcare professionals, which is consistent with previous research [23,25,26].

Furthermore, our study results on participants’ intentions to remain in their current medical speciality indicate valuable insights regarding Romanian dental professionals. The responses to whether they would choose the same speciality again provide an understanding of their satisfaction with their current medical speciality. Most respondents (59.42%) indicated they would choose the same speciality again, suggesting a relatively high level of satisfaction with their career choice during the COVID-19 pandemic. However, a notable proportion of respondents (21.74%) expressed uncertainty about their career future, indicating potential doubts or concerns about their current path. This uncertainty may be related to various factors, including burnout, work-related stress, or other personal and professional considerations, as previous research suggests [14,21,27,28].

Furthermore, 18.84% of respondents in our sample group expressed a clear intention to change their medical speciality if given a chance again. The higher mean age of respondents in this group (39.92 years) compared with those who would choose the same speciality again (38.75 years) may suggest that age and accumulated experience could be factors influencing the decision to change profession, which is consistent with the results of the burnout literature in the healthcare field, where the intention to leave the profession is influenced by several factors, apart from self-reported burnout dimensions. Thus, burnout and the intention to leave the medical profession are associated with adverse work conditions, low workforce density, and a lack of personal resources [29,30,31,32].

Our study results and the consistency with the data from the existing literature suggest that, especially after the COVID-19 pandemic, the importance of addressing burnout in healthcare professionals to retain a skilled and committed workforce remains very high. Significant levels of burnout and the intention to change professions have an important impacts on patient care and organisational stability [30]. Healthcare institutions should prioritise interventions and support systems aimed at reducing burnout and promoting well-being among dental healthcare professionals [33,34,35,36].

Understanding the impact of burnout on the intention to change professions can help healthcare organisations tailor strategies to retain healthcare practitioners and foster a positive work environment. By providing resources such as counselling, stress management programs, and flexible work arrangements, healthcare organisations can support their employees in coping with burnout and making informed decisions about their careers [37,38].

Our study findings regarding the intention to change profession align with previous research in healthcare settings. A previous study investigated burnout and career satisfaction among physicians and found that physicians experiencing burnout were more likely to consider leaving their profession [39]. Similarly, previous studies suggest a correlation between burnout and turnover intentions among other medical specialities. In one study, burnout was a significant predictor of healthcare practitioners leaving their current position or the profession altogether [28].

Prior to the COVID-19 pandemic, the existing literature suggested that high burnout appears to be associated with some sociodemographic and job-related characteristics, particularly younger age, male gender, an absence of vocation, and unwillingness to reselect dentistry as a job. [2]. Our findings suggest that after the pandemic, our respondents reporting change-in-profession intentions are more likely to be women, consistent with recent study results [3,23,40]. The Hoff et al. study [23] suggests that female doctors are generally more likely to experience burnout than male doctors, particularly regarding the emotional Exhaustion dimension of burnout, as data from 43 studies were part of the review. Similarly, recent research on paediatric dentists in the United States reveals that they may be more susceptible to occupational burnout and/or depression due to the chronic stress associated with providing child dental treatment and an increasing proportion of females in the dental profession [3].

Our findings are consistent with previous research in healthcare settings, including studies on physicians, which have shown that burnout is associated with a higher likelihood of considering leaving one’s current profession [24,27,28,39]. Moreover, other studies in various medical specialities have also demonstrated correlations between burnout and turnover intentions among healthcare professionals [41]. These studies collectively support that burnout can significantly affect healthcare professionals’ career decisions. It may contribute to workforce instability, pushing healthcare professionals to their limits, as a Spanish study suggests for primary care physicians in Catalonia [42].

The alignment of our study’s findings with the existing literature highlights the importance of addressing burnout in healthcare professionals to retain a skilled and committed workforce [43]. High levels of burnout impact individual well-being and can have serious consequences for patient care and organisational stability. Dentists and other healthcare professionals experiencing burnout may be at risk of limited job satisfaction, less productivity, and, finally, considering renouncing their current position or the profession altogether and experiencing poor physical and mental health issues [9,14,28].

Healthcare institutions should prioritise implementing effective interventions and support systems to reduce the negative impacts of burnout and its possible impact on the intention to change profession [5,43,44,45,46,47]. Healthcare practitioners who are experiencing burnout and the difficulties that come with it can benefit greatly from counselling and mental health resources. Programmes for stress management can help with resilience development and general well-being enhancement. Offering flexible work choices, such as part-time work or job sharing, may help healthcare professionals maintain a better work–life balance and experience less burnout.

Furthermore, retaining healthcare workers and fostering their career happiness depends on promoting a positive and encouraging work environment [45]. A sense of value and involvement inside a healthcare facility can be encouraged by promoting open communication, offering chances for professional growth, and acknowledging the work of healthcare practitioners [33,43,48].

Our study provides valuable insights into the distribution of burnout levels across gender sub-scales among Romanian dental healthcare professionals. Female participants reported higher levels of emotional Exhaustion (EE) and Depersonalisation (D) than males. However, there were no substantial differences in Personal Accomplishment levels between genders.

These findings have implications for addressing burnout in dental healthcare professionals, emphasising the need to recognise and address gender differences in burnout experiences. Healthcare organisations and policymakers should consider implementing gender-specific interventions and support systems to mitigate burnout and promote well-being among dental healthcare professionals. Additionally, further research is warranted to explore the factors contributing to the gender differences observed in emotional Exhaustion and Depersonalisation levels. By addressing burnout effectively, we can enhance the overall well-being of medical healthcare professionals and improve the quality of patient care.

Our study has several limitations. The sample size of 69 Romanian dentists may not be fully representative of all Romanian dental professionals, considering diversity in practice settings, which may affect the generalisability of the present results. The collected data rely on a self-reported questionnaire, which might allow for a response bias, underreporting, or overreporting burnout dimensions based on factors such as social desirability. As for the generalisability of the results, this study focuses on dental healthcare providers in Romania during the COVID-19 crisis and the findings may not be applicable to dentists in other countries and different healthcare systems. Also, a cross-sectional design while identifying associations cannot establish causality, whereas longitudinal studies provide a more robust insight into the causal relationship between burnout dimensions and intentions to leave one’s profession.

## 5. Conclusions

Our study findings suggest gender differences in burnout experience and perception, with a higher proportion of females reporting high levels of emotional Exhaustion compared with male dental professionals. Depersonalisation was minimal in both categories and Personal Accomplishment data provided less clear gender differences in our study. The expressed intentions to change profession in our sample group was positively corelated with emotional Exhaustion, bearing a moderate positive effect on the desire to change medical speciality. Healthcare institutions should consider interventions and support systems to address burnout and promote well-being among dental healthcare professionals.

## Figures and Tables

**Table 1:** Demographic data.

	Mean Age	CI		Std. Dev.	Std. Er.	Min	Max	Q25	Q75
		−95%	+95%						
Female 56 (81.15%)	38.78	36.50	41.06	8.49	1.13	26	62	31	45
Male 13 (18.84%)	38.84	33.61	44.07	8.65	2.40	26	53	32.50	43.50
All	38.79	36.76	40.83	8.46	1.01	26	62	32	44

**Table 2:** Gender distribution of subscale scores.

Subscale		Female	Male	Chi-Test	TOTAL
Emotional Exhaustion (EE)	High	40 (71.42%)	6 (46.15%)	0.024	46 (66.66%)
	Moderate	11 (19.64%)	2 (15.38%)		13 (18.84%)
	Low	5 (8.92%)	5 (38.46%)		10 (14.49%)
Depersonalisation (D)	High	12 (21.42%)	2 (15.38%)	0.805	14 (20.28%)
	Moderate	15 (26.78%)	3 (23.07%)		18 (26.08%)
	Low	29 (51.78%)	8 (61.53%)		37 (53.62%)
Personal Accomplishment (PA)	High	16 (28.57%)	4 (30.76%)	0.983	20 (28.98%)
	Moderate	14 (25.00%)	3 (23.07%)		17 (24.63%)
	Low	26 (46.42%)	6 (46.15%)		32 (46.37%)

**Table 3:** The intention to change profession correlated with burnout subscales.

		EE	D	PA	Change in Profession
EE	Pearson Correlation	1	0.648 **	−0.385 **	0.307 *
	Sig. (2-tailed)		0.000	0.001	0.010
	N	69	69	69	69
D	Pearson Correlation	0.648 **	1	−0.702 **	0.163
	Sig. (2-tailed)	0.000		0.000	0.181
	N	69	69	69	69
PA	Pearson Correlation	−0.385 **	−0.702 **	1	−0.085
	Sig. (2-tailed)	0.001	0.000		0.486
	N	69	69	69	69
Change in profession	Pearson Correlation	0.307 *	0.163	−0.085	1
	Sig. (2-tailed)	0.010	0.181	0.486	
	N	69	69	69	69

* Correlation is significant at the 0.05 level (2-tailed); ** Correlation is significant at the 0.01 level (2-tailed).

**Table 4:** The relationship between intention to change profession and EE, D, and PA predictors.

Model		Unstandardised Coefficients		Standardised Coefficients	t	Sig.
		B	Std. Error	Beta		
1	Constant	0.788	0.344		2.288	0.025
	EE	0.117	0.045	0.338	2.612	0.011
	D	−0.043	0.062	−0.117	−0.696	0.489
	PA	0.094	0.061	0.221	1.543	0.128

## Data Availability

The data presented in this study are available on request from the corresponding author. The data are not publicly available due to licence constraints.
